# Does duration of physical activity bouts matter for adiposity and metabolic syndrome? A cross-sectional study of older British men

**DOI:** 10.1186/s12966-016-0361-2

**Published:** 2016-03-15

**Authors:** Barbara J. Jefferis, Tessa J. Parsons, Claudio Sartini, Sarah Ash, Lucy T. Lennon, S. Goya Wannamethee, I-Min Lee, Peter H. Whincup

**Affiliations:** UCL Department of Primary Care & Population Health, UCL Medical School, Rowland Hill Street, London, NW3 2PF UK; UCL Physical Activity Research Group, London, UK; Brigham and Women’s Hospital, Harvard Medical School, Boston, MA 02215 USA; Population Health Research Institute, St George’s University of London, Cranmer Terrace, London, SW17 0RE UK

**Keywords:** Physical activity, Sedentary behaviour, Metabolic syndrome, BMI, Accelerometer, Bouts, Cohort, Older adults, Men

## Abstract

**Background:**

Older adults have low physical activity(PA) and high sedentary behaviour(SB) levels. We investigate how total volume and specific patterns of moderate to vigorous PA(MVPA), light PA(LPA) and SB are related to adiposity and metabolic syndrome (MS). Then, with reference to physical activity guidelines which encourage MVPA in bouts > =10 min and avoiding “long” sedentary bouts, we investigate whether accumulating PA and SB in bouts of different defined durations are differently associated with these outcomes.

**Methods:**

Cross-sectional study of men (71–91 years) recruited in UK primary care centres. Nurses made physical measures (weight, height, bio-impedance, blood pressure) and took fasting blood samples. 1528/3137 (49 %) surviving men had ≥3 valid days (≥600 min) accelerometer data. 450 men with pre-existing chronic disease were excluded. 1009/1078 (93.6 %) had complete covariate data.

**Results:**

Men (*n* = 1009, mean age 78.5(SD 4.7) years) spent 612(SD 83), 202(SD 64) and 42(SD 33) minutes in SB, LIPA and MVPA respectively. Each additional 30 min/day of SB and MVPA were associated with 0.32 (95 % CI 0.23, 0.40)Kg/m^2^ higher Body Mass Index (BMI) and −0.72(−0.93, −0.51) lower BMI Kg/m^2^ respectively. Patterns for waist circumference (WC), fat mass index (FMI), fasting insulin and MS were similar. MVPA in bouts lasting <10 min or ≥10 min duration were not associated differently with outcomes. In models adjusted for total MVPA, each minute accumulated in SB bouts lasting 1–15 min was associated with lower BMI −0.012 kg/m^2^, WC −0.029 cm, and OR 0.989 for MS (all *p* < 0.05), and coefficients for LPA bouts 1–9 min were very similar in separate models adjusted for total MVPA. Minutes accumulated in SB bouts 1–15 min and LPA bouts 1–9 min were correlated, *r* = 0.62.

**Conclusions:**

Objectively measured MVPA, LPA and SB were all associated with lower adiposity and metabolic risk. The beneficial associations of LPA are encouraging for older adults for whom initiating MVPA and maintaining bouts lasting ≥10 min may be particularly challenging. Findings that short bouts of LPA (1–9 min) and SB (1–15 min), but that all MVPA, not just MVPA accumulated in bouts ≥10 min were associated with lower adiposity and better metabolic health could help refine older adult PA guidelines.

## Background

To date epidemiologic studies of physical activity (PA) in relation to health outcomes have mostly concentrated on estimating associations with volume of PA of a specified intensity such as moderate or vigorous intensity activity (MVPA). Yet UK guidelines specify that PA and sedentary behaviour(SB) should be accumulated in specific patterns; 150 min of MVPA per week should be accumulated in bouts lasting 10 min or more, and “long” sedentary periods should be avoided [1]. However large epidemiologic studies using objective measures of PA and SB have not simultaneously tested what duration of SB might be detrimental, and whether shorter bouts of MVPA e.g., <10 min duration, or bouts of lighter activity may be beneficial [2]. These questions are particularly pertinent to older adults who are the least active and most sedentary age group, with the lowest levels of adherence to current MVPA guidelines [3]. The use of objective measures of PA and SB providing minute by minute data can help identify specific beneficial patterns of bouts of activity of specified durations and clarify the importance of activity intensity more accurately than using questionnaire based measures of PA.

Previous studies have demonstrated that higher levels of self-reported physical activity (PA) and lower levels of self-reported sedentary behaviour (SB) (primarily television viewing) are related to lower waist circumference (WC), body mass index (BMI) and prevalence of the metabolic syndrome (MS) in cross-sectional [4, 5] and longitudinal studies [6–8]. However, recent cross-sectional studies using objectively measured PA report stronger associations for self-reported than objective measures of SB [5, 9, 10]. Differences could be due to measurement error or recall bias in self reports (for which there is evidence particularly among older adults [11, 12]). Television viewing time is commonly used to measure SB, yet it is only a subset of total SB, defined as time spent expending less than 1.5 METS whilst sitting or reclining [13]. Furthermore, residual confounding by social background or unhealthy behaviours including dietary habits [14], could partly explain the associations between television viewing and adverse outcomes that are not observed for total daily sedentary time. Objectively measuring PA and SB can reduce measurement and recall biases and account for all activities during waking hours and therefore clarify how different intensities and patterns of activities are related to important health outcomes including adiposity and markers of CVD risk and metabolic health.

We use a large sample of community-dwelling older men to investigate associations between total volume of objectively measured MVPA, LPA and SB with markers of metabolic health; WC, BMI, fat mass index (FMI), fasting serum insulin and metabolic syndrome. Uniquely, we then investigate whether bouts of MVPA, LPA and SB of different specified durations are associated with all of these outcomes, in order to understand whether only activity volume is important, or if pattern of accumulation also matters in older adults.

## Methods

### Measures

#### Sample

Data are from the British Regional Heart Study an on-going prospective, population-based cohort. 7735 men recruited from primary care centres in 24 British towns in 1978–80 were followed-up [15]. In 2010–2012, 3137 survivors were invited to a physical examination and an accelerometer study. The National Research Ethics Service (NRES) Committee London provided ethical approval. Participants provided informed written consent to the investigation, which was performed in accordance with the Declaration of Helsinki. Men attended a clinic where nurses made measurements: height using a stadiometer (Harpenden); weight and fat mass (bioimpedance) in light indoor clothing with a Tanita body composition analyser (BC-418), or on Tanita scales (weight only) if the participant had a pacemaker or defibrillator. Fat Mass Index (FMI) was calculated by standardising to height squared. Waist circumference (WC, cm) was measured at the mid-point between the iliac crest and the lower edge of the ribs. A fasting blood sample was collected; insulin (mmol/l) was assayed by ELISA. Insulin, triglycerides, high density lipoprotein cholesterol (HDLC) and glucose (all mmol/l) were adjusted for length of fasting and time of day. Systolic and diastolic blood pressure were measured twice (Omron recorder, mmHg) and the average of 2 readings used. Metabolic Syndrome (MS) was defined using the updated NCEP criteria [16].

#### Accelerometer data

Men wore a GT3X accelerometer (Actigraph, Pensacola, Florida) on a belt over the right hip for 7 days during waking hours, removing it for swimming or bathing. Accelerometer data were processed using standard methods described previously [17]. Counts per minute (CPM) were calculated from movements registering on the vertical axis. Non-wear time was identified and excluded using the commonly used R package “Physical Activity” [18]. Periods of continuous zeros lasting more than 90 min were assigned as non-wear time; short spells of non-zero counts lasting up to 2 min during the 90 min period were allowed as non-wear time if no activity counts were detected during both the 30 min before and after that interval, to reflect the possibility of artefactual monitor movements (e.g., due to accidental movement of the monitor being disturbed while left on a table). This means that any non-zero counts except the allowed short interval of up to 2 min are considered as wear time. Analyses included participants with ≥3 valid days (≥600 min wear time), a conventional requirement to estimate usual PA level [19–21]. Standard threshold values of CPM developed for older adults [22]: <100 for SB (<1.5 MET),100–1040 for light activity (LPA) (1.5- < 3 MET) and >1040 for MVPA,(≥3 MET) were used to categorise the number of minutes/day spent in each intensity level.

#### Questionnaire data

Men self-reported current cigarette and alcohol consumption, whether they lived alone or with others and number of hours of night time sleep, any doctor diagnosis of heart attack, heart failure, stroke (with symptoms lasting >24 h) or type 2 diabetes and use of medications including antihypertensives (British National Formulary (BNF) codes 2.2.1, 2.2.8, 2.4, 2.5.5 and 2.6.2.) or statins (BNF code 2.12). Social class based on longest held occupation reported at study entry (1978–80) was categorised as manual or non-manual [23]. Region of residence (1978–80) was grouped into Scotland, North, Midlands and South of England.

#### Availability of data and materials

Data may be requested from http://www.ucl.ac.uk/pcph/research-groups-themes/brhs-pub/tools.

### Statistical methods

Q-Q plots indicated that the number of daily minutes in SB and LPA were normally distributed and minutes of MVPA were right-skewed. Within quartiles of daily minutes of SB and MVPA descriptive statistics (% and number for categorical, or mean and standard error for continuous variables) were calculated for demographic characteristics, objective measures of PA and SB and metabolic markers. The exposures investigated were firstly: total daily minutes of SB, LPA and MVPA. Secondly, total daily minutes spent in bouts of (a) SB lasting 1–15 min, 16–30 min, 31–60 min, ≥61 min, (b) LPA lasting 1–9 min and ≥10 min and (c) bouts of MVPA lasting 1–9 min and ≥10 min. Bouts were consecutive minutes within the CPM thresholds for SB, LPA or MVPA, without a “grace period” outside the threshold. The MVPA bouts were based on the 10 min recommendation from current PA guidelines [1]; however, in the absence of recommendations, the duration of bouts of SB and LPA were chosen based on their distributions. Like MVPA, LPA was split into bouts < or ≥10 min, as most LPA bouts were short. The duration of SB bouts varied more, permitting four categories of adequate group size covering a range of durations. Pearson’s correlation coefficients were calculated between the categories of bouts of PA and SB.

Linear and quadratic models and Generalised Additive Models (GAM) were used to investigate whether associations between total SB, LPA and MVPA and each outcome were linear. Associations between each PA measure and the continuous outcomes (BMI, WC, FMI and blood insulin level) were assessed using linear regression models and logistic regression models were used for metabolic syndrome (yes/no). All models were adjusted for measurement related factors (season of accelerometer wear, average accelerometer wear time (minutes/day), region of residence), and confounders (age, social class, living alone, smoking status and alcohol consumption). The first models assessed independent associations of total minutes of each exposure intensity (MVPA, LPA and SB, Models 1a,b and c respectively) with each outcome. The next models included the bouts of activity in categories: Model 2a = minutes/day of MVPA in bouts of 1–9 and ≥10 min, Model 2b = minutes/day LPA in bouts 1–9 and ≥10 min, Model 2c = minutes/day SB in bouts 1–15, 16–30, 31–60 and ≥61 min. Wald tests evaluated whether the coefficients for categories of minutes of bouts of different durations differed. If the associations with the outcomes did not differ between the bout categories, then the single variable (total minutes/day at that intensity) was used for the final models. To assess the independence of associations of the each intensity with each outcome, SB and MVPA (model 3), and MVPA and LPA (model 4) were mutually adjusted. Models including SB were not also adjusted for LPA to avoid problems of collinearity (LPA and SB were correlated, *r* = −0.63, *P* < 0.001). Models were not adjusted simultaneously for total minutes of SB, LPA and MVPA because the sum of the three has to equal the total wear time (which was included in the models), so models would not converge. Complete case analysis was used.

As sensitivity analyses, models were re-run using (i) z-score transformations of total minutes (in SB, LPA and MVPA) to evaluate whether the associations with the outcome variables were of similar magnitude with each activity intensity (ii) z-score transformations for outcomes (BMI, WC, FMI and insulin) to evaluate whether the associations with the PA and SB variables were of similar magnitude with each outcome (iii) square-root transformation of minutes of MVPA (correcting for right skew) (iv) additional adjustment for commonly used medicines; statins and anti-hypertensives (v) additional adjustment for total self-reported night time sleep.

Men with pre-existing illness (a doctor diagnosis of heart attack, heart failure, stroke (with symptoms lasting >24 h), type 2 diabetes or fasting blood glucose ≥7 mmol/l) were excluded from analyses.

## Results

3137 survivors were invited, of whom 1655 (53 %) agreed to participate, 1566 (50 %) returned an accelerometer with data, and 1528 (49 %) men had sufficient data (≥600 min/day wear time on ≥3 days). 450 men with one or more pre-existing illness were excluded (heart failure *n* = 31, stroke *n* = 50, CHD *n* = 202 and diabetes *n* = 258 cases). Of the remaining 1078, 1009 had data on adiposity and co-variates, mean age was 78.5 (SD 4.7) years. The mean daily accelerometer wear time was 856 (SD 67) minutes, of which 612 (SD 83) minutes were spent in SB, 202 (SD 64) minutes in LPA and 42 (SD 33) minutes in MVPA. The distribution of minutes of MVPA was right skewed, median 33 min (inter quartile range 17–57).

Table 1 presents the correlates of SB and PA. Associations were graded across quartiles of SB; the more sedentary men were older, more likely to live alone, smoke cigarettes, consume less alcohol, and spend less time in LPA and MVPA. They had higher BMI, WC, FMI and fasting insulin than the least sedentary men. Similarly graded associations, in the opposite direction were observed over quartiles of MVPA.

**Table 1:** Characteristics of 1078 men without pre-existing diabetes, CVD or heart failure, % (*n*) or mean (SE)

	Quartile of SB (minutes/day)						
	1	2	3	4			
	(295 – <564)	(≥564 – <618)	(≥618 – <674)	(≥674)	*P* (trend)	*N*	*N* missing
*N* ^a^	287^a^	275^a^	268^a^	248^a^		1078	
Age (years), mean (SE)	77.3 (0.2)	77.8 (0.3)	79.1 (0.3)	79.9 (0.3)	<0.0001	1078	0
Non-manual social class, % (*n*)	53.9 (154)	59.5 (163)	53.2 (141)	50.0 (123)	0.18^b^	1071	7
Living alone, % (*n*)	14.6 (41)	15.1 (41)	19.9 (53)	24.4 (60)	0.01^b^	1064	14
Smoking, % (*n*)	1.8 (5)	1.1 (3)	4.6 (12)	7.0 (17)	0.001^c^	1062	16
Alcohol (units per week), mean (SE)	6.9 (0.5)	6.7 (0.5)	6.3 (0.5)	5.7 (0.4)	0.06	1047	31
% day spent in SB, mean (SE)	61.5 (0.4)	70.5 (0.3)	75.3 (0.3)	80.7 (0.4)	<0.0001	1078	0
SB (mins/day), mean (SE)^d^	509 (3)	591 (1)	644 (1)	720 (2)	<0.0001	1078	0
LPA (mins/day), mean (SE)^d^	256 (2)	209 (1)	184 (1)	153 (2)	<0.0001	1078	0
MVPA (mins/day), mean (SE)^d^	66.8 (1.9)	42.4 (1.4)	31.6 (1.1)	22.0 (1.0)	<0.0001	1078	0
BMI (kg/m^2^), mean (SE)^d^	26.3 (0.2)	26.7 (0.2)	26.9 (0.2)	27.3 (0.3)	<0.0001	1068	10
WC (cm), mean (SE)^d^	97.0 (0.5)	98.4 (0.60)	99.8 (0.6)	100.3 (0.8)	<0.0001	1072	6
FMI (kg/m^2^), mean (SE)^d^	9.8 (0.2)	10.1 (0.2)	10.5 (0.2)	10.7 (0.2)	<0.0001	1009	69
Insulin (mmol/l), mean (SE)^d^	7.8 (0.3)	9.3 (0.4)	9.6 (0.4)	8.5 (0.3)	<0.0001	1010	68
MS, % (*n*)	18.5 (48)	20.6 (51)	24.2 (56)	25.5 (55)	0.23	954	124
	Quartile of MVPA (minutes/day)						
	1	2	3	4			
	(0.4 – <3.1)	(≥3.1 – <30.8)	(≥30.8 – <53.5)	(≥53.5)	*P* (trend)	*N*	*N* missing
*N* ^a^	226^a^	270^a^	286^a^	296^a^		1078	
Age (years), mean (SE)	8.4 (0.3)	78.6 (0.3)	78.1 (0.2)	76.6 (0.2)	<0.0001	1078	0
Non-manual social class, % (*n*)	48 (109)	55 (148)	55 (158)	57 (166)	0.25^b^	1071	7
Living alone, % (*n*)	21 (47)	19 (52)	19 (53)	15 (43)	0.28^b^	1064	14
Smoking, yes % (*n*)	6.3 (14)	4.5 (12)	1.4 (4)	2.4 (7)	0.013^c^	1062	16
Alcohol (units per week), mean (SE)	5.2 (0.5)	6.1 (0.5)	6.9 (0.5)	7.2 (0.5)	0.001	1047	31
% time spent Sedentary, mean (SE)	81.3 (0.4)	75.0 (0.3)	70.1 (0.3)	62.7 (0.4)	<0.0001	1078	0
SB(mins/day), mean (SE)^d^	672 (4)	636 (3)	605 (3)	551 (4)	<0.0001	1078	0
LPA (mins/day), mean (SE)^d^	148 (3)	190 (3)	216 (3)	242 (3)	<0.0001	1078	0
MVPA (mins/day), mean (SE)^d^	7.2 (0.2)	22.2 (0.3)	41.5 (0.4)	85.3 (1.6)	<0.0001	1078	0
BMI (kg/m^2^), mean (SE)^d^	27.5 (0.3)	27.3 (0.2)	26.5 (0.2)	26.0 (0.2)	<0.0001	1068	10
WC (cm), mean (SE)^d^	101.3 (0.8)	100.5 (0.6)	98.1 (0.6)	96.0 (0.5)	<0.0001	1072	6
FMI, (kg/m^2^), mean (SE)^d^	10.9 (0.2)	10.7 (0.2)	10.1 (0.2)	9.6 (0.2)	<0.0001	1009	69
Insulin (mmol/l), mean (SE)^d^	9.4 (0.5)	9.9 (0.4)	8.5 (0.3)	7.7 (0.3)	<0.0001	1010	68
MS, % (*n*)	27.1 (52)	28.7 (68)	20.6 (52)	14.0 (38)	<0.0001	954	124

*SB* sedentary behaviour, *LPA* light activity, *MVPA* moderate and vigorous physical activity, *BMI* body mass index, *WC* waist circumference, *FMI* fat mass index, *MS* metabolic syndrome

^a^maximum N in quartile, varies slightly with missing covariate data

^b^Pearson chi square test

^c^Fisher’s exact test

^d^adjusted for average daily accelerometer wear time, season of wear, region of residence, age

Table 2 presents the distribution of categories of bouts of MVPA, LPA and SB. 75 % of MVPA and 89 % of LPA was accrued in bouts lasting under 10 min. 37 % of SB was accrued in bouts lasting 1–15 min and 18 % in bouts lasting ≥61 min. Table 3 presents correlations between PA outcomes: total SB minutes were inversely correlated with LPA (*r* = −0.63) and with MVPA (*r* = −0.55), both *p* < 0.0001. Total MVPA minutes were positively correlated with minutes spent in bouts of LPA 1–9 min (*r* = 0.52) and with minutes spent in short sedentary bouts 1–15 min (*r* = 0.11) but inversely correlated with minutes spent in longer sedentary bouts (lasting longer than 16 min) e.g., *r* = −0.38 for bouts lasting ≥61 min, (all *p* < 0.01). Correlations between LPA in bouts of 1–9 min and the short (1–15) and long (≥61) bouts of SB were in the same direction as for MVPA, but much stronger e.g., *r* = 0.59 and −0.67, respectively. Higher numbers of minutes spent in short sedentary bouts (1–15 min) were inversely and weakly correlated with total SB minutes (*r* = −0.08) but more strongly inversely correlated with minutes spent in longer SB bouts (e.g., *r* = −0.63 for SB bouts ≥61 min).

**Table 2:** Distribution of bouts of moderate to vigorous PA, light PA and SB, *n* = 1078

	%
Bouts of MVPA (>1040 CPM)	
1–9 min	75
> = 10 min	25
Bouts of LPA (100–1040 CPM)	
1–9 min	89
> = 10 min	11
Bouts of SB (<100 CPM)	
1–15 min	37
16–30 min	21
31–60 min	24
> = 61 min	18

*MVPA* moderate and vigorous physical activity, *LPA* light activity, *SB* sedentary behaviour

**Table 3:** Correlations (Pearson’s r) between moderate to vigorous PA, light PA and SB, *n* = 1078

	Total MVPA (mins/day)	Total LPA (mins/day)	Total SB (mins/day)	LPA 1–9 min bouts (mins/day)	LPA > =10 min bouts (mins/day)	SB 1–15 min bouts (mins/day)	SB 16–30 min bouts (mins/day)	SB 31–60 min bouts (mins/day)
Total MVPA (mins/day)	1							
Total LPA (mins/day)	0.48*	1						
Total SB (mins/day)	−0.55*	−0.63*	1					
MVPA mins in bouts 1–9 mins	0.90*	0.58*	−0.59*					
MVPA mins in bouts 10+ mins	0.77*	0.16*	−0.29*					
LPA mins in bouts 1–9 mins	0.52*	0.97*	−0.59*	1				
LPA mins in bouts 10+ mins	0.22*	0.77*	−0.54*	0.59*	1			
SB mins in bouts 1–15 mins	0.11**	0.54*	−0.08**	0.62*	0.16*	1		
SB mins in bouts 16–30 mins	−0.17*	−0.15*	0.39*	−0.10**	−0.23*	0.23*	1	
SB mins in bouts 31–60 mins	−0.35*	−0.56*	0.64*	−0.55*	−0.39*	−0.39*	0.19*	1
SB mins in bouts ≥61 mins	−0.38*	−0.64*	0.58*	−0.67*	−0.35*	−0.63*	−0.27*	0.26*

*MVPA* moderate and vigorous physical activity, *LPA* light activity, *SB* sedentary behaviour

*p < 0.0001

**p < 0.01

Table 4 presents associations between total minutes of MVPA, LPA and SB with each health outcome in Models 1a, 1b and 1c; MVPA was inversely associated with BMI, WC, FMI, insulin level and metabolic syndrome. Coefficients in Table 4 are expressed per minute of MVPA, LPA and SB. Multiplying by 30, each additional 30 min of MVPA per day, was associated with 0.72 (95 % CI 0.93, 0.51) Kg/m^2^ lower BMI and OR 0.68 (95 % CI 0.56, 0.82) for metabolic syndrome (Model 1a). Associations with LPA were in the same direction, but effect sizes were smaller (Model 1b). The coefficients for LPA were almost identical in magnitude to the coefficients for SB (model 1c), but in the opposite direction (as expected given the inverse correlation). Each additional 30 min of SB per day was associated with an increase in BMI similar in size to the reduction associated with 30 min of LPA; 0.32(0.23, 0.40) Kg/m^2^ and OR for metabolic syndrome was 1.13 (95 % CI 1.05, 1.21) (Model 1c). Similar patterns were seen for WC, FMI, and insulin levels. Additional adjustment for WC fully attenuated the associations between MVPA or SB with insulin (data not presented). The associations between daily minutes spent in bouts MVPA, LPA and SB of different durations are presented in Models 2a-2c respectively. In Model 2a coefficients for associations between MVPA and outcomes appeared slightly smaller for bouts <10 min compared to > =10 min, but Wald tests did not find evidence that they differed (*p* = 0.3), so subsequent analyses use total minutes of MVPA. Minutes of LPA accumulated in bouts of 1–9 min were inversely associated with all of the outcomes, and Wald tests suggested these coefficients differed from those for minutes of LPA in bouts of ≥10 min for BMI and insulin (Model 2b). Time in sedentary bouts lasting 1–15 min was not associated with any outcome, but more time spent in longer bouts (31–60 and ≥61 min) was positively associated with each outcome and Wald tests indicated that associations of each outcome with minutes in bouts 31–60 or ≥61 min differed from minutes in bouts lasting 1–15 min (Model 2c). Hence subsequent models include daily minutes of LPA in bouts of 1–9 and ≥10 min and SB in bouts of 1–15, 16–30, 31–60 and ≥61 min.

**Table 4:** Associations between moderate to vigorous PA, light PA and SB (minutes/day) with body composition, fasting serum insulin, [β (95 % CI)] and metabolic syndrome [OR (95 % CI)]

	BMI (kg/m^2^)		WC (cm)		FMI (kg/m^2^)		Insulin (mmol/l)		MS	
	*N* = 1019		*N* = 1023		*N* = 966		*N* = 962		*N* = 907	
	β	(95 % CI)	β	(95 % CI)	β	(95 % CI)	β	(95 % CI)	OR	(95 % CI)
Model 1; Total minutes/day										
Model 1a										
Total MVPA mins/day	**−0.024**	**(−0.031,−0.017)**	**−0.080**	**(−0.101,−0.060)**	**−0.020**	**(−0.026,−0.014)**	**−0.024**	**(−0.036,−0.012)**	**0.987**	**(0.981,0.993)**
Model 1b										
Total LPA mins/day	**−0.011**	**(−0.015,−0.007)**	**−0.034**	**(−0.045,−0.023)**	**−0.009**	**(−0.013,−0.006)**	**−0.009**	**(−0.015,−0.002)**	0.997	(0.994,1.000)
Model 1c										
Total SB mins/day	**0.011**	**(0.008,0.013)**	**0.034**	**(0.025,0.042)**	**0.009**	**(0.006,0.011)**	**0.009**	**(0.004,0.014)**	**1.004**	**(1.002,1.006)**
Model 2; Minutes/day in bouts										
Model 2a										
MVPA mins/day in bouts 1–9 mins	**−0.020**	**(−0.030,−0.009)**	**−0.068**	**(−0.099,−0.036)**	**−0.016**	**(−0.025,−0.007)**	−0.018	(−0.036,0.000)	0.991	(0.982,1.000)
MVPA mins/day in bouts ≥10 mins	**−0.031**	**(−0.046,−0.016)**	**−0.100**	**(−0.144,−0.057)**	**−0.026**	**(−0.039,−0.013)**	**−0.034**	**(−0.059,−0.010)**	**0.980**	**(0.966,0.995)**
Coefficients equal to each other^a^		*p* = 0.30		*p* = 0.30		*p* = 0.29		*p* = 0.35		*p* = 0.3
Model 2b										
LPA mins/day in bouts 1–9 mins	**−0.018**	**(−0.024,−0.012)**	**−0.048**	**(−0.065,−0.030)**	**−0.013**	**(−0.018,−0.008)**	**−0.022**	**(−0.032,−0.012)**	**0.994**	**(0.990,0.999)**
LPA mins/day in bouts ≥10 mins	0.011	(−0.003,0.026)	0.006	(−0.037,0.049)	0.001	(−0.011,0.013)	**0.032**	**(0.008,0.056)**	1.004	(0.993,1.015)
Coefficients equal to each other^a^		0.002		0.05		0.09		*p* = 0.0005		*p* = 0.2
Model 2c										
SB mins/day in bouts 1–15 mins	0.000	(−0.007,0.007)	0.009	(−0.012,0.030)	0.002	(−0.004,0.008)	−0.003	(−0.014,0.009)	0.998	(0.992,1.003)
SB mins/day in bouts 16–30 mins	0.006	(−0.001,0.013)	0.022	(−0.000,0.043)	0.006	(−0.000,0.012)	**0.017**	**(0.005,0.030)**	1.001	(0.996,1.007)
SB mins/day in bouts 31–60 mins	**0.007**	**(0.001,0.012)**	**0.033**	**(0.017,0.049)**	**0.010**	**(0.006,0.015)**	**0.010**	**(0.001,0.019)**	**1.005**	**(1.001,1.010)**
SB mins/day in bouts ≥61 mins	**0.011**	**(0.007,0.015)**	**0.029**	**(0.017,0.041)**	**0.006**	**(0.003,0.010)**	0.003	(−0.004,0.010)	1.002	(0.998,1.005)
Coefficients equal to each other^a^		*p* = 0.0002		*p* = 0.02		*p* = 0.04		*p* = 0.03		*p* = 0.03
Model 3; MVPA and SB mutually adjusted										
MVPA mins/day in bouts ≥1 mins	**−0.023**	**(−0.033,−0.012)**	**−0.071**	**(−0.102,−0.040)**	**−0.016**	**(−0.025,−0.007)**	**−0.026**	**(−0.043,−0.008)**	**0.982**	**(0.973,0.992)**
SB mins/day in bouts 1–15 mins	**−0.012**	**(−0.021,−0.003)**	**−0.029**	**(−0.055,−0.002)**	−0.006	(−0.014,0.002)	**−0.017**	**(−0.031,−0.002)**	**0.989**	**(0.982,0.996)**
SB mins/day in bouts 16–30 mins	−0.001	(−0.009,0.007)	0.001	(−0.023,0.024)	0.002	(−0.005,0.008)	0.010	(−0.004,0.023)	0.996	(0.990,1.002)
SB mins/day in bouts 31–60 mins	−0.001	(−0.007,0.005)	0.009	(−0.010,0.028)	0.005	(−0.000,0.011)	0.002	(−0.009,0.012)	1.000	(0.995,1.005)
SB mins/day in bouts ≥61 mins	0.003	(−0.002,0.008)	0.005	(−0.011,0.021)	0.001	(−0.003,0.006)	−0.006	(−0.015,0.003)	0.996	(0.992,1.000)
Model 4; MVPA and LPA mutually adjusted										
MVPA mins/day in bouts ≥1 mins	**−0.017**	**(−0.025,−0.010)**	**−0.064**	**(−0.087,−0.041)**	**−0.015**	**(−0.022,−0.008)**	**−0.017**	**(−0.029,−0.004)**	**0.988**	**(0.981,0.995)**
LPA mins/day in bouts 1–9 mins	**−0.012**	**(−0.019,−0.006)**	**−0.025**	**(−0.044,−0.006)**	**−0.007**	**(−0.013,−0.002)**	**−0.017**	**(−0.028,−0.006)**	0.998	(0.993,1.003)
LPA mins/day in bouts ≥10 mins	0.007	(−0.008,0.021)	−0.010	(−0.052,0.033)	−0.003	(−0.015,0.009)	**0.028**	**(0.004, 0.052)**	1.002	(0.990,1.013)

Coefficients adjusted for average daily accelerometer wear time, season of wear, region of residence, age, social class, living alone, tobacco and alcohol use

*BMI* body mass index, *WC* waist circumference, *FMI* fat mass index, *MS* metabolic syndrome, *MVPA* moderate and vigorous physical activity, *LPA* light activity, *SB* sedentary behaviour. Coefficients in bold are statistically significant at 5 % level

^a^Wald test

Model 3 mutually adjusts MVPA with SB and model 4 mutually adjusts MVPA with LPA to test whether associations with one activity intensity are independent of the other intensity. Model 3 is mutually adjusted for MVPA and bouts of SB; compared to unadjusted coefficients in Model 1a and 2c coefficients for daily minutes of MVPA were little changed whereas short bouts of SB 1–15 min were significant. Multiplying coefficients by 30, each additional 30 min of MVPA was associated with lower BMI, WC, FMI, insulin (−0.69 kg/m^2^, −2.13 cm, −0.48 kg/m^2^, −0.78 mmol/L respectively) and OR 0.58 for metabolic syndrome, all *p* < 0.05. Each additional 30 min accumulated in short bouts of SB (lasting 1–15 min) was associated with lower BMI, WC and insulin (−0.36 kg/m^2^, −0.87 cm, and −0.51 mmol/L respectively) and OR 0.71 for metabolic syndrome, all *p* < 0.05. In Model 4, mutual adjustment of total MVPA and bouts of LPA slightly weakened the coefficients for MVPA and LPA (compared to model 1a and 2b), but both remained inversely associated with each of the outcomes, and, with the exception of insulin the coefficients for MVPA suggested stronger associations with each outcome than for LPA. Short bouts (1–9 min) of LPA and (1–15 min) of SB were positively correlated (*r* = 0.62, *p* < 0.0001, Table 3) and so the coefficients for associations of LPA 1–9 min with health outcomes (Model 4, Table 4) were very similar to those for SB bouts 1–15 min (Model 3).

Sensitivity analyses using z-scores of exposures (total daily minutes of SB, LPA and MVPA), found that the relative effect size of 1-SD change in each activity scores of SB, LPA and MVPA were of similar magnitude, but coefficients for MVPA tended to be slightly larger than for LPA, particularly for insulin and WC (data not presented). Models using z-scores of outcomes (BMI, WC FMI and insulin), found that the relative effect size of associations of MVPA, LPA and SB levels and bouts (as in Table 3) with BMI, WC and FMI were of similar magnitude (data not presented), although associations with insulin were somewhat weaker. Sensitivity analyses using square root transformed total minutes of MVPA (to correct for skewness), did not alter the main findings, so untransformed results are presented for ease of interpretation. When models were run with additional adjustment for (i) statins and anti-hypertensives (taken by 40.2 and 49.9 % of men respectively) or (ii) total self-reported night time sleep (mean 6.9, SD 1.3 h/night), results and conclusions were unchanged.

## Discussion

In this large cross-sectional study of community-dwelling older men, higher levels of MVPA and lower levels of SB were associated with better metabolic health in terms of WC, BMI, FMI, insulin level and metabolic syndrome. To our knowledge this is the first large epidemiologic study to investigate how bouts of accelerometer-measured MVPA, LPA and SB of different specified durations are associated with all of these markers of adiposity and metabolic health in older adults. We found that minutes spent in MVPA, whether accrued in bouts lasting over 10 min (as per current guidelines) or lasting less than 10 min, were associated with lower levels of adiposity and lower risk of metabolic syndrome, indicating that accumulating all MVPA, regardless of bout duration was beneficial. In this sample, 89 % of LPA was accumulated in bouts lasting <10 min and minutes spent in these bouts were associated with lower levels of adiposity and lower risks of metabolic syndrome, but not as strongly as minutes spent in MVPA. The associations with LPA persisted after adjusting for MVPA, suggesting that bouts of light activity could confer important additional benefits regardless of participation in MVPA. LPA bouts lasting 1–9 min were correlated (*r* > 0.6), with SB bouts lasting 1–15 min and inversely correlated with long SB bouts lasting over one hour (*r* < −0.6), suggesting that men who frequently broke up LPA also frequently broke up SB. In models of SB bouts adjusted for MVPA, breaking up sedentary time into more short sedentary bouts (1–15 min) was associated with lower levels of adiposity, fasting insulin and metabolic syndrome, although the effect size for MVPA was larger than for short bouts of SB.

### Strengths and limitations

Key strengths are the novel investigation of bouts of MVPA, LPA and SB of different specified lengths which goes beyond existing studies which had previously just investigated number breaks in SB [24, 25] or limited numbers of bouts [26–29] rather than a range of specific durations, and had not tested different bouts of MVPA, LPA and SB in one study. Our definition of bouts did not include a “grace period” if activity dropped below, or raised above the cut point threshold for a minute, but required that bouts be consecutive minutes between specified thresholds. So if for example a participant was within the light activity range (100–1040 CPM) but then exceeded the MVPA threshold (>1040) for one minute this would have ended the LPA bout and hence could have underestimated the impact of bouts of LPA on outcomes. The study also benefits from objective measures both of exposures (PA and SB), and of a wide range of outcomes (measured height, weight and bioimpedance and fasting blood samples). The sample is community-dwelling adults rather than “at risk” clinical groups, so results should be generalizable to wider older adult populations, although perhaps not to younger ages or women, who have different patterns of PA [17] and adiposity. The 49 % response rate to the accelerometer study compares favourably to other studies of older adults: 21 % [20], 43 % [19] and in the Health Survey for England 37 % women and 48 % men over 75 years had > =4 days with valid data [30]. We used an accelerometer which is validated for measuring low levels of energy expenditure but lacked good inclinometer data to assess whether subjects were standing or sitting during periods of <100 CPM. Hence we cannot be certain that each minute <100 CPM was sitting or reclining rather than standing; however the mean CPM during these minutes was 9 CPM suggesting that very little movement during time classed as sedentary and it was unlikely to include a lot of standing time (particularly given our age range 70–90 years). We based our choice of cut points of best current evidence, but among older adults with a range of functional abilities, there may be variability in the CPM cut point which represents exactly 3 METS, which could impact on the observed results. The cross-sectional design limits our ability to make causal inferences about the observed associations and longitudinal studies will be required to assess the direction of causation between patterns of PA and SB and metabolic health.

### Comparison with other studies

#### Total MVPA, LPA and SB

Overall the associations between total amounts of objectively measured MVPA and SB with adiposity and fasting insulin were in the expected direction and fit with prior studies [9, 25, 31]. The coefficients for the beneficial associations of 30 min of MVPA with each outcome were much larger the coefficients for 30 min of SB time. This was expected since 30 min of MVPA is a much larger proportion of total daily MVPA time than 30 min of SB is of total daily SB time, and is therefore a much bigger relative change. When MVPA and SB were standardised to their standard deviations, the coefficients are broadly similar to each other for BMI, WC, FMI and a little smaller for insulin. We also investigated LPA which is less studied; indeed current PA guidelines offer no recommendations about LPA because of lack of evidence about its associations with health outcomes [1, 2]. LPA may be particularly important in older adults for whom MVPA may be difficult to initiate and maintain in sustained bouts. We found significant associations between LPA and all metabolic outcomes, which fits with the reduced odds of metabolic syndrome in middle aged Japanese adults associated with LPA [32]. In our study total time spent in LPA and SB were inversely correlated, and their associations with health outcomes were of similar magnitude and in the opposite direction. Thus men who spent less time in SB did more LPA, and hence less SB or more LPA were both beneficially associated with health outcomes. Our findings that LPA was not as strongly associated as MVPA with adiposity or insulin, fits with cross-sectional data from NHANES, where a similar pattern of associations between LPA and WC and fasting insulin was observed [33]. There are fewer studies of LPA in relation to measures of adiposity other than BMI; however a study of British 60–64 year olds reported that higher levels of objectively measured SB and lower LPA and MVPA were associated with higher FMI, although associations with LPA were attenuated on adjustment for MVPA [34]. A systematic review found cross-sectional studies of very low and moderate quality supporting positive associations between total SB time and BMI and WC [8]. There are conflicting results about whether SB is associated with particular patterns of regional fat deposition; in a small study in a high-risk population, objectively measured SB adjusted for MVPA was positively associated with heart, liver and visceral fat, but not subcutaneous or whole body fat [35]. In contrast, in a healthy population of adults (mean age 66 years), when MVPA and SB were mutually adjusted, only MVPA was associated with pericardial fat [36].

#### Bouts of MVPA, LPA and SB

Our investigation of the importance of bouts of activity of MVPA, LPA and SB of several different defined lengths in relation to a wide range of metabolic markers in older adults is novel and provides important data to inform guidelines. In our older adult population associations with each outcome appeared somewhat stronger for the MVPA in bouts ≥10 compared to <10 min, but they were not significantly different, suggesting no additional benefits of accumulating MVPA in bouts over and above the total time in MVPA, for BMI, WC, FMI and metabolic syndrome. This fits with conclusions of analyses of NHANES data in relation to BMI [28, 37], WC [27, 38], triceps and subscapular skinfolds [38] and insulin [27] and to metabolic syndrome [29] in Canadian Health Measures study, although these studies used different accelerometer protocols and comparator groups and younger samples. However, other findings using NHANES suggested that associations of minutes of MVPA in ≥10 min bouts with BMI [26, 38] and WC [26] were stronger for than for minutes of MVPA accumulated in bouts lasting <10 min, or total activity level [27], but comparability is limited as they use very different cut-points to define MVPA [26, 27, 38]. A recent intervention trial also reported greater weight loss among participants with more MVPA in bouts of ≥10 min and higher levels of LPA [39]. We did not find evidence for associations between minutes in bouts of LPA lasting ≥10 min and the outcomes studied, perhaps because in this population only 10 % of LPA was accumulated in bouts lasting ≥10 min, so confidence intervals are wider than for estimates for bouts of 1–9 min. However, an association might be detected in populations where LPA is accumulated in longer bouts. More frequent bouts of LPA lasting 1–9 min (and their correlate, more frequent bouts of SB lasting 1–15 min) were associated with lower adiposity and insulin levels. The direction of these effects fit with studies investigating number of sedentary breaks [31]. Among middle aged and younger adults, fewer breaks in SB (reflecting more prolonged sedentary bouts on average) are associated with higher WC and BMI [25, 31, 40] whereas insulin was related to longer total SB, but not to breaks in SB [31].

## Conclusions

Objectively measured MVPA, LPA and SB were all associated with metabolic risk in later life. The beneficial associations of LPA with metabolic health are encouraging for older adults for whom initiating MVPA and maintaining bouts lasting ≥10 min may be particularly challenging. For the first time, we have quantified what patterns of accumulation of activity and SB are associated with adiposity and metabolic health in a large-scale study of older adults. Our findings that all MVPA was beneficial, irrespective of being accumulated in bouts lasting < or ≥10 min, and that breaking SB into short bouts of 1–15 min, and its correlate LPA in bouts 1–9 min were also beneficially associated with the metabolic outcomes are relevant to clinicians advising patients about daily activity, and to policy-makers refining current SB guidelines.

## References

[1] Chief Medical Officers of England SWaNI (2011). Start active, stay active. A report on physical activity for health from the four home countries’ Chief Medical Officers.

[2] Physical Activity Guidelines Advisory Committee. Physical Activity Guidelines Advisory Committee Report. Washington, DC;U.S. Department of Health and Human Services. 2008.

[3] Chaudhury M, Roth M, Craig R, Mindell J (2008). Physical activity. Health survey for England 2006. Volume 1. Cardiovascular disease and risk factors in adults.

[4] Edwardson CL, Gorely T, Davies MJ, Gray LJ, Khunti K, Wilmot EG, Yates T, Biddle SJ (2012). Association of sedentary behaviour with metabolic syndrome: a meta-analysis. PLoS ONE.

[5] Stamatakis E, Hamer M, Tilling K, Lawlor DA (2012). Sedentary time in relation to cardio-metabolic risk factors: differential associations for self-report vs accelerometry in working age adults. Int J Epidemiol.

[6] Hu FB, Li TY, Colditz GA, Willett WC, Manson JE (2003). Television watching and other sedentary behaviors in relation to risk of obesity and type 2 diabetes mellitus in women. JAMA.

[7] Wijndaele K, Healy GN, Dunstan DW, Barnett AG, Salmon J, Shaw JE, Zimmet PZ, Owen N (2010). Increased cardiometabolic risk is associated with increased TV viewing time. Med Sci Sports Exerc.

[8] de Rezende LF, Rey-Lopez JP, Matsudo VK, do Carmo LO (2014). Sedentary behavior and health outcomes among older adults: a systematic review. BMC Public Health.

[9] Celis-Morales CA, Perez-Bravo F, Ibanez L, Salas C, Bailey ME, Gill JM (2012). Objective vs. self-reported physical activity and sedentary time: effects of measurement method on relationships with risk biomarkers. PLoS One.

[10] Atienza AA, Moser RP, Perna F, Dodd K, Ballard-Barbash R, Troiano RP, Berrigan D (2011). Self-reported and objectively measured activity related to biomarkers using NHANES. Med Sci Sports Exerc.

[11] Van Cauwenberg J, Van Holle V, De Bourdeaudhuij I, Owen N, Deforche B (2014). Older adults’ reporting of specific sedentary behaviors: validity and reliability. BMC Public Health.

[12] Gennuso KP, Matthews CE, Colbert LH (2015). Reliability and validity of 2 self-report measures to assess sedentary behavior in older adults. J Phys Act Health.

[13] Sedentary Behaviour Research Network (2012). Letter to the editor: standardized use of the terms “sedentary” and “sedentary behaviours”. Appl Physiol Nutr Metab.

[14] Scully M, Dixon H, Wakefield M (2009). Association between commercial television exposure and fast-food consumption among adults. Public Health Nutr.

[15] Walker M, Whincup PH, Shaper AG (2004). The British Regional Heart Study 1975–2004. Int J Epidemiol.

[16] Grundy SM, Cleeman JI, Daniels SR, Donato KA, Eckel RH, Franklin BA, Gordon DJ, Krauss RM, Savage PJ, Smith SC (2005). Diagnosis and management of the metabolic syndrome - An American Heart Association/National Heart, Lung, and Blood Institute Scientific Statement. Circulation.

[17] Jefferis BJ, Sartini C, Lee IM, Choi M, Amuzu A, Gutierrez C, Casas JP, Ash S, Lennon L, Wannamethee SG (2014). Adherence to physical activity guidelines in older adults, using objectively measured physical activity in a population-based study. BMC Public Health.

[18] Choi L, Liu Z, Matthews C, Buchowski MS (2011). Physical activity: process physical activity accelerometer data. (0.1-1). 2011.

[19] Harris TJ, Owen CG, Victor CR, Adams R, Cook DG (2009). What factors are associated with physical activity in older people, assessed objectively by accelerometry?. Br J Sports Med.

[20] Davis MG, Fox KR, Hillsdon M, Sharp DJ, Coulson JC, Thompson JL (2011). Objectively measured physical activity in a diverse sample of older urban UK adults. Med Sci Sports Exerc.

[21] Hart TL, Swartz AM, Cashin SE, Strath SJ (2011). How many days of monitoring predict physical activity and sedentary behaviour in older adults?. Int J Behav Nutr Phys Act.

[22] Copeland JL, Esliger DW (2009). Accelerometer assessment of physical activity in active, healthy older adults. J Aging Phys Act.

[23] Office of Population Censuses and Surveys (1970). 1970 classification of occupations.

[24] Gennuso KP, Gangnon RE, Thraen-Borowski KM, Colbert LH (2015). Dose–response relationships between sedentary behaviour and the metabolic syndrome and its components. Diabetologia.

[25] Henson J, Yates T, Biddle SJ, Edwardson CL, Khunti K, Wilmot EG, Gray LJ, Gorely T, Nimmo MA, Davies MJ (2013). Associations of objectively measured sedentary behaviour and physical activity with markers of cardiometabolic health. Diabetologia.

[26] Strath SJ, Holleman RG, Ronis DL, Swartz AM, Richardson CR (2008). Objective physical activity accumulation in bouts and nonbouts and relation to markers of obesity in US adults. Prev Chronic Dis.

[27] Wolff-Hughes DL, Fitzhugh EC, Bassett DR, Churilla JR (2015). Total activity counts and bouted minutes of moderate-to-vigorous physical activity: relationships with cardiometabolic biomarkers using 2003–2006 NHANES. J Phys Act Health.

[28] Salvo D, Torres C, Villa U, Rivera JA, Sarmiento OL, Reis RS, Pratt M (2015). Accelerometer-based physical activity levels among Mexican adults and their relation with sociodemographic characteristics and BMI: a cross-sectional study. Int J Behav Nutr Phys Act.

[29] Clarke J, Janssen I (2014). Sporadic and bouted physical activity and the metabolic syndrome in adults. Med Sci Sports Exerc.

[30] Craig R, Mindell J, Hirani V (2009). Health survey for England 2008. Physical activity and fitness. Summary of key findings.

[31] Healy GN, Matthews CE, Dunstan DW, Winkler EA, Owen N (2011). Sedentary time and cardio-metabolic biomarkers in US adults: NHANES 2003–06. Eur Heart J.

[32] Kim J, Tanabe K, Yokoyama N, Zempo H, Kuno S (2013). Objectively measured light-intensity lifestyle activity and sedentary time are independently associated with metabolic syndrome: a cross-sectional study of Japanese adults. Int J Behav Nutr Phys Act.

[33] Buman MP, Winkler EAH, Kurka JM, Hekler EB, Baldwin CM, Owen N, Ainsworth BE, Healy GN, Gardiner PA (2014). Reallocating time to sleep, sedentary behaviors, or active behaviors: associations with cardiovascular disease risk biomarkers, NHANES 2005–2006. Am J Epidemiol.

[34] Bann D, Kuh D, Wills AK, Adams J, Brage S, Cooper R (2014). Physical activity across adulthood in relation to fat and lean body mass in early old age: findings from the Medical Research Council National Survey of Health and Development, 1946–2010. Am J Epidemiol.

[35] Henson J, Edwardson CL, Morgan B, Horsfield MA, Bodicoat DH, Biddle SJ, Gorely T, Nimmo MA, McCann GP, Khunti K (2014). Associations of sedentary time with fat distribution in a high-risk population. Med Sci Sports Exerc.

[36] Hamer M, Venuraju SM, Urbanova L, Lahiri A, Steptoe A (2012). Physical activity, sedentary time, and pericardial fat in healthy older adults. Obesity (Silver Spring).

[37] Fan JX, Brown BB, Hanson H, Kowaleski-Jones L, Smith KR, Zick CD (2013). Moderate to vigorous physical activity and weight outcomes: does every minute count?. Am J Health Promot.

[38] Loprinzi PD, Cardinal BJ (2013). Association between biologic outcomes and objectively measured physical activity accumulated in > = 10-minute bouts and < 10-minute bouts. Am J Health Promot.

[39] Jakicic JM, Tate DF, Lang W, Davis KK, Polzien K, Neiberg RH, Rickman AD, Erickson K (2014). Objective physical activity and weight loss in adults: the step-up randomized clinical trial. Obesity (Silver Spring).

[40] Healy GN, Dunstan DW, Salmon J, Cerin E, Shaw JE, Zimmet PZ, Owen N (2008). Breaks in sedentary time: beneficial associations with metabolic risk. Diabetes Care.

